# Nature Relatedness of Recreational Horseback Riders and Its Association with Mood and Wellbeing

**DOI:** 10.3390/ijerph17114136

**Published:** 2020-06-10

**Authors:** Gabriele Schwarzmüller-Erber, Harald Stummer, Manfred Maier, Michael Kundi

**Affiliations:** 1 Department of General Practice and Family Medicine, Center for Public Health, Medical University Vienna, Kinderspitalgasse 15/1, 1090 Vienna, Austria; manfred.maier@meduniwien.ac.at; 2 Health Sciences, University of Applied Sciences FH Campus Wien, Favoritenstrasse 226, 1100 Vienna, Austria; 3 Institute for Management & Economics in Health Care, UMIT, 6060 Hall i.T., Austria; harald.stummer@umit.at; 4 Faculty of Business, University Seeburg Castle, Seeburgstraße 8, 5201 Seekirchen/Wallersee, Austria; 5 Department of Environmental Health, Center for Public Health, Medical University Vienna, Kinderspitalgasse 15/1, 1090 Vienna, Austria; michael.kundi@meduniwien.ac.at

**Keywords:** nature relatedness, recreational horseback riding, dog walking, subjective wellbeing, mood

## Abstract

Connectedness to nature and nature contact can provide many benefits to humans, like stress reduction, recovery from illness, and increased positive emotions. Likewise, recreational horseback riding is a widespread sports activity with the potential to enhance physical and psychological health. Yet, the influence of connectedness to nature on the wellbeing of older aged recreational horseback riders has not been investigated so far. The aim of the present study therefore was to explore the relationship between nature relatedness and physical, psychological and social wellbeing and happiness. The study sample was composed of Austrian recreational horseback riders aged 45 years and older, who were compared with dog owners and people without pets (*n* = 178). We found significantly higher nature relatedness, significantly higher overall wellbeing and a significantly better mood rating in recreational horseback riders compared to people without pets and similar scores compared to dog owners. Physical wellbeing is correlated with overall nature relatedness in horseback riders and dog owners, but no correlation was found in people without pets. A structural equation model shows a direct relationship between nature relatedness and mood in horseback riders and an indirect relationship through pet attachment in dog owners. The results suggest the activity with horses and dogs in nature environments is a source of wellbeing, enjoyment, self-confidence and social contacts.

## 1. Introduction

Horse riding and dog ownership seem to be associated with both nature relatedness and physical activity. However, there is a lack of evidence on the association of nature relatedness, pet attachment, physical activity and physical, psychological and social wellbeing. 

### 1.1. Physical Activity and its Association with Wellbeing 

Health promotion by means of increased physical activity is maybe the most promising strategy to counteract age-related decreased quality of life. Not only cardiorespiratory fitness [1,2], physical functioning [1,3], bone health [4] and balance [5], but also wellbeing in general [6] can be affected by regular activities or resistance training even at an older age. In particular, movement attributable to horseback riding is associated with positive physical and mental improvements such as muscle strengthening [7,8], trunk stabilization [9] and mental wellbeing [10], both in children and adults. 

Although it is widely accepted that health-related quality of life and health itself are influenced by participation in sports [11], around 23% of adults worldwide do not achieve the recommended activity levels, with a minimum 150 min of medium intensity per week [12]. To reach the recommended activity levels, exercise with pets, dog walking or horseback riding could be beneficial. The influence of dogs on activity levels is well documented [13] for different age groups, such as children [14,15], adults [16,17] and the elderly [18,19,20]. However, for recreational horseback riding no such evidence exists in the elderly.

### 1.2. Nature Relatedness and its Association with Wellbeing and Perceived Health

Nature relatedness (NR) describes a close feeling with nature and different levels of connectedness with the natural world [21], which means it describes the cognitive, affective, and physical connection of people with nature [21,22]. Its origin is based among others on the Biophilia hypothesis [23] of E.O. Wilson, who proposed that people have an instinctive, intrinsic demand to attach to others and other living things [24], and to nature in general [22,25]. The motivation to spend time in nature and its influence on wellbeing is based on this attachment assumption [22,25].

In historical dimensions, only very recently have humans spent most of their time in built environments [26,27] and time spent in nature is decreasing. Different studies have investigated the impact of nature or activity in natural environments on wellbeing [28,29,30]. Living close to green spaces is a motivator for physical activity [31]. 

Activities in nature are associated with stress reduction [27], wellbeing and happiness [26,32]. The higher the connectedness to nature, the higher the life satisfaction and the higher the happiness or positive emotion [33,34]. Furthermore, time spent in nature may also be used to increase physical activity [35]. 

Walking through natural environments results in higher physical fitness [36]. Compared to indoor sports or outdoor sports in built environments, physical activity in nature leads to better sleep quality and emotional wellbeing [37] and a healthier lifestyle [35]. Immune function could be improved through time in outdoor environments as well [38,39]. Natural environments compared to city areas increase parasympathetic nerve activity, whereas sympathetic nerve activity decreases [39,40]. Additionally, an increase in natural killer cell activity after walking in forests has been observed [38,41]. 

In addition to physical wellbeing, psychological and emotional wellbeing, expressed as happiness, are positively influenced by contact with nature [42,43,44]. Physical activity in natural environments can cause increased mental wellbeing [45]. Therefore, nature-based physical activity programs, such as therapeutic horseback riding, can improve mental health [46]. Feelings like calmness or refreshment are significantly higher after activity in natural environments compared to activities in a city [39]. Walking or running in nature can result in less sadness [43], tension, confusion and anger [45], while cognitive capacity [47] as well as energy, such as vitality and enthusiasm, can increase [43,45]. Community gardening among the elderly [48] and children [49] can lead to physical and social benefits [50]. People visiting green spaces and having a stronger relatedness to nature more frequently experience better social cohesion [28], and activities in green spaces stimulate personal development, e.g., increased body appreciation [33]. An increased social contact frequency due to activity in a natural environment has been reported too [33,34].

### 1.3. Pet Attachment and its Association with Wellbeing

The relationship with animals can be seen as a third pillar for health care. Bowlby’s (1975) attachment theory [51] proposed not only relationships to humans, as originally described, but also to animals [20,52]. According to this theory people can develop a bond to pets and profit from it in various dimensions, e.g., through the presence of a dog in therapeutic settings [53]. It is not only a relationship to the animal; people need the animal to fill a void in life or self-esteem for a positive outcome, and this is called attachment [54]. 

In several studies pet owners indicate high levels of attachment to their pets both in adults [55], in children [56], but also in mentally ill patients [57] and elderly people [20,58,59]. Regardless of the animal species, attachment to dogs [16,60,61,62], cats [20], and horses [56] are observed. As published before, our study showed that horse ownership was related to overall pet attachment, but riders had significantly lower scores and sub-scores compared to dog owners [63]. Overall, pet attachment was associated with mental and social wellbeing. By contrast, no association was found with physical wellbeing neither in riders nor in dog owners [63]. On the other hand, studies with dog owners indicated a correlation between pet attachment and physical wellbeing [64,65]. Pets induce increased social interaction [66] and quality of life [67] and have a positive influence on mental health [68,69]. However, despite all the positive effects, negative consequences of pet ownership, such as injuries [70,71,72,73] or zoonoses [67,74], are also possible.

### 1.4. The Present Study

Although studying this triad, the influence of connectedness to nature, the influence of pet attachment and the influence of physical activity on wellbeing and mood in middle- and older-aged (45+) recreational horseback riders, seems to be a straightforward research path given the above mentioned evidence, so far no such study has been conducted. Overall, there is a lack of studies concerning recreational horseback riding in the elderly. Therefore, the present study aims to investigate the influence of these three factors on wellbeing and mood (predominant emotion or feelings) of recreational horseback riders in comparison to dog owners. 

We reported about the relationship between pet attachment and riders’ and dog owners´ wellbeing [63]. Based on these findings, we hypothesized that recreational horseback riders aged 45+, show nature relatedness (NR) scores, pet attachment (PA) and activity levels comparable to dog owners, whom have been studied more extensively. Secondly, we hypothesized that these parameters (nature relatedness, pet attachment and activity levels) were associated with physical, psychological and social wellbeing and happiness (mood). 

## 2. Materials and Methods 

### 2.1. Participants

The recruitment strategy has been described before [63] and included conducting a search for riders with the help of the Equestrian Association of Austria (OEPS), which is dedicated to different riding disciplines and has about 48,500 members [75]. The search for dog owners was conducted with the help of the Working Dog Sports Association of Austria (OEGV), with about 55,000 members [76], which is dedicated to education and training of dogs and owners. All riding clubs and dog associations received accurate information about the study. People without pets, as controls, were found through a call in a weekly newspaper and announcements in shops in the same regions the people with pets came from. One hundred and eighty-four riders and dog owners [63] and 56 people without pets followed the call to participate in the study and were contacted via mail or phone.

To assess inclusion and exclusion criteria, a screening conversation via face to face or telephone was conducted with all individuals identified. Subjects were eligible, if they met the following inclusion criteria: at least 45 years or older, being a long-standing recreational horseback rider or dog owner, or do not own any animal (controls), and being an Austrian citizen. Exclusion criteria were health impairments making horseback riding, dog walking or household and sports activities impossible, unable to answer a questionnaire in German, or owning both dogs and horses. For controls, of course, owning a pet was an exclusion criterion. 

All selected participants agreed to spend one hour answering the questionnaire and signed a written informed consent form. 

This study was carried out according to the Helsinki declaration of 1964 [77] and according to data safety guidelines. Due to a waiver it was exempted from ethics approval (IRB of lower Austria: date of submission 18 July 2014, date of waiver 22 July 2014).

### 2.2. Data Collection and Management

All selected persons received the 14-page questionnaire and the informed consent form via e-mail or postal mail. They were requested to fill in the questionnaire directly after performing their hobby, which means after riding, after dog playing (we used the wording ”working” for dog owners, who participated in courses with their dogs), or after performing the other hobby for people without pets, and to return them via e-mail or postal mail. The questionnaires were numbered consecutively and manually transferred to the database. After a check for completeness and signature on the consent form, all questionnaires were numbered for anonymization and manually transferred to SPSS statistics version 24 (IBM Corp., Armonk, NY, USA). 

### 2.3. Questionnaires

Questionnaires were prepared in German language with five self-reporting sections (habitual wellbeing, nature relatedness, pet attachment (for rider and dog owner), wellbeing and mood during/after riding, dog walking or performing one’s hobby, and socio-demographic characteristics and activities in household, sports and with the horse or dog). Parts not relevant for this article have been described before [63].

The 21-item nature relatedness scale, translated into German, developed by Nisbet, Zelenski, and Murphy [21], was used to assess connectedness to nature. It detects nature enthusiasts, self-identified conservationists [78]. Statements are rated using a five-point Likert scale, from 1 = disagree strongly to 5 = strongly agree. Nature relatedness scores were calculated by averaging items representing each subscale: the “nature-related experience” score (6 items), the “nature-related-self” score (8 items) and the “nature-related perspective” score (7 items) [21]. The higher the scores, the higher the relatedness to nature [78]. The questionnaire has a good internal consistency α = 0.87 for the total score, α = 0.80 for nature-related experience, α = 0.84 for nature-related self and α = 0.66 for nature-related perspective [78]. The current study shows good internal consistency α = 0.80 for the total score, nature-related self α = 0.72, nature-related perspective α = 0.54 and nature-related experience α = 0.64. 

To evaluate pet attachment, positive and negative aspects of relationships, the German translated Life-Impact Scale PALS-35 [79] with 35 items was used. Pet attachment scores were calculated averaging items representing each subscale: “love” (17 items), “regulation of emotions” (9 Items), “personal growth” (5 items), and “negative impact” (4 items reversed scored) [79]. It uses a five-point Likert scale, from 1 = “not at all” to 5 = “very much” (Cronbach’s alpha = 0.907) [79]. The current study shows a Cronbach’s alpha of 0.934 for the total PALS with 35 items, α = 0.894 for the “love” factor, α = 0.877 for the “regulation” factor, α = 0.811 for the “personal growth” factor and α = 0.485 for “negative impact” factor [63].

Physical, psychological and social wellbeing of participants were measured by the “Fragebogen zum habituellem Wohlbefinden (habitual subjective wellbeing scale)” FAHW 12-questionnaire [80]. Items are scored on a five-point Likert scale (Cronbach’s α: 0.85) [80]. General habitual wellbeing (FAHW12) scores were calculated as the sum of responses to the items representing each subscale with a score range from 1 = “certainly not to” 5 = “yes, exactly.” Items with negative content were subtracted [80]. The current study shows a good internal consistency (total FAHW 12: α = 0.80; physical wellbeing: α = 0.82; psychological wellbeing: α = 0.63; social wellbeing: α = 0.53) [63].

To evaluate the influence of recreational horseback riding, dog walking (influence of training) or any other hobby in controls on wellbeing, a self-designed questionnaire, which asked for the influence of these activities on personal feelings (28 items for physical, psychological and mental wellbeing), was used. This part of the whole questionnaire was filled in directly after performing the hobby (riding, “playing with dog”, performing other hobby) for two time-periods: the current time (“after activity”) and in retrospective (“during activity”). The items were dichotomous and positive answers were summed up for measuring wellbeing (physical, psychological and social) during and after the activity (Cronbach’s α = 0.80 for the total score and Cronbach’s α = 0.56–0.66 for the subscales) [63].

After the self-reported assessment of the influence of training (riding, dog playing, other hobby) on subjective wellbeing, respondents reported their mood (emotions using to a mood barometer) by rating a single Kunin (face) item [81] ranging, according to Andrews and Withey (1976) [80,82], from A = 7 points to G = 1 point. All participants were asked, “Which face illustrates best how you felt during and feel now after riding/dog playing or dog walking/performing your hobby?” [63].


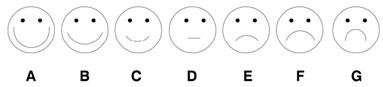


Demographic data of participants, such as gender, age, height and weight, educational status and living conditions (living with a partner or not), were assessed in the last part of the 14-page questionnaire. The answers were used in general linear models to address possible confounding and comparison of groups. Additionally, participants specified their sports and household activities, which were later transferred to metabolic equivalent hours per week, according to the Compendium of Physical Activities [83].

### 2.4. Statistical Analyses

Descriptive statistics were used to characterize the study groups. For metric data we report mean +/− SEM (standard error of mean) unless otherwise stated; for categorical data, percentages are presented, and chi-square tests and Mann–Whitney tests were applied for group comparison. 

FAHW 12-scores were calculated as the sum of responses to the items with a score ranging from −18 to 25 and the sum of responses to the items representing each subscale, ranging from −8 to 18 for physical, from −3 to 11 for psychological and from −8 to 8 for social wellbeing. Higher scores indicate higher wellbeing [63,80]. 

To evaluate subjective activity-related wellbeing (during and after horseback riding, dog playing/working or performing other hobby), the sum scores were computed for each subscale: physical, ranging from 0 to 10, psychological and social wellbeing, ranging from 0 to 9. After the activity sum scores reach for physical from 0 to 10; for performing other hobbys), the sum scores were computed for each subscale: physical, ranging from 0 to 10, psychological from 0 to 9 and for social from 0 to 5. Higher values indicate higher wellbeing during or after the activity [63]. 

For the description of mood during/after the activity we summarized AB for “very good”, CD for “good”, EF for “bad” and G for “very bad” mood. 

To calculate pet attachment scores, the mean of responses to the items ranging from 1 to 5 was computed for the total scale and for its subscales “love,” “regulation,” “personal growth” and “negative impact.” According to Cromer and Barlow, higher scores indicate high pet attachment [63,79]. 

To calculate nature relatedness scores, the mean of responses to the items ranging from 1 to 5 was computed for the total scale and for the three subscales “nature-related experience”, “nature-related self” and “nature-related perspective.“ According to Nisbeth et al. [21], the higher the scores, the higher the nature relatedness. 

For comparison of nature relatedness, wellbeing and pet attachment scores between groups, a general linear model adjusted for gender, age, BMI (calculated from weight and height), marital status (fixed or no partnership) and education (more or less than 12 years) was applied. 

Normal distribution of residuals was assessed by Kolmogorov–Smirnov tests with Lilliefors’ corrected *p*-values [84] and homogeneity of variances by Levene’s tests [85]. 

To determine if pet attachment levels influenced the pattern of wellbeing, a structural equation model was applied to the data separately for riders and dog owners. 

All analyses were performed using SPSS version 24 (IBM Corp., Armonk, NY, USA) except for structural equitation modelling, which was performed using SEPATH within Statistica 10 (StatSoft, OK, USA). For all tests statistical significance was set at 5% (*p* < 0.05).

## 3. Results

Of 240 eligible persons, 183 people returned the questionnaire and the signed informed consent form by post or e-mail (response rate 76%). Five questionnaires were excluded due to missing information or missing signature. Overall, 67 recreational horseback riders, 57 dog owners and 54 people without pets, older than 44 years of age fulfilled the inclusion criteria and were included in this study (total *n* = 178).

### 3.1. Sample Characteristics

The majority of participants were females (88.1% of riders, 63.2% of dog owners, 59.3% of those without pets) (*p* < 0.001). Age of riders ranged from 45 to 82 years (mean +/− SD: 55.5 +/− 8.4 years), of dog owners from 45 to 80 years (58.7 +/− 10.1 years) and of people without pets from 45 to 92 years (61.7 +/− 11.6 years) and differed significantly across groups (*p* = 0.013). Additionally, body weight and BMI differed (weight: *p* = 0.010, BMI: *p* = 0.005). Average BMI for riders was 23.8 +/− 3.8 kg/m^2^; (mean+/− SD: range: 18–36 kg/m^2^), of dog owners (26.6 +/− 5.2 kg/m^2^; range: 18–43 kg/m^2^) and of controls (BMI: 25.4 +/− 4.3 kg/m^2^; range: 18–41 kg/m^2^). Overall, 23.9% of riders, 19.3% of dog owners and 31.5% of people without pets lived without a partner. Education was similar for the three groups: 92.5% of riders, 93% of dog owners and 85.2% of controls reported 12 years or more education.

### 3.2. Nature Relatedness

**Figure 1:**
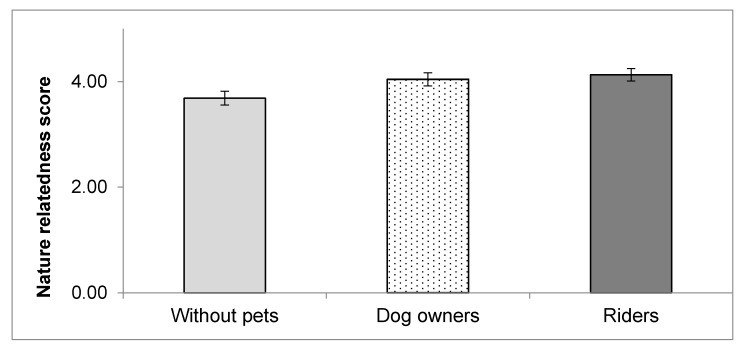
Mean and 95% confidence intervals of nature relatedness scores by study group.

We found significantly higher NR scores of riders compared to people without pets (*p* < 0.001) but similar scores compared to dog owners (*p* = 0.325). Mean score of riders was 4.13 +/− 0.06 SEM, of dog owners 4.04 +/− 0.07 SEM and of controls without pets 3.69 +/− 0.07 SEM. People without pets showed less relatedness to nature relatedness scores than riders and dog owners (Figure 1). Covariates age, gender, education, living status and BMI did not confound these results.

Additionally, sub-scales of NR riders differed significantly from controls but not from dog owners (Table 1).

### 3.3. Physical Activity

Neither sports activities (*p* = 0.142) (rider: 10.0 +/− 2.2, dog owner 8.8 +/− 2.6, without pets: 9.8 +/− 1.9 MET-h/week) nor household activities (*p* = 0.094) differ significantly between groups (riders 16.9 +/− 3.3 MET-h/week, dog owners 15.5 +/− 2.1 MET-h/week, without pets 11.9 +/− 2.2 MET-h/week). Additionally, no statistically significant difference was found between riders and dog owners (*p* = 0.253) in overall activities in MET-h/week (riders: 107.4 +/− 62.0, dog owners: 137.4 +/− 116.7 vs. MET-h/week), including activities with the pets.

### 3.4. Pet Attachment

As published in the previous paper, horseback riders had a similar attachment to their pets (3.76 +/− 0.634 SEM) compared to dog owners (3.91 +/− 0.613 SEM) (*p* = 0.053). The covariates age, sex, BMI, marital status and educational status were considered as potential confounders [63].

Dog owners reported significantly (*p* = 0.023) higher pet attachment “love” scores (mean 4.22 +/− 0.09 SEM) compared to riders (3.94 +/− 0.08 SEM). “Regulation of emotions” was also significantly lower (*p* = 0.009) in recreational horseback riders (3.23 +/− 0.11 SEM) compared to dog owners (3.69 +/− 0.13 SEM). However, no significant differences concerning the sub-scores “personal growth” (riders: 3.44 +/− 0.12 SEM; dog owners: 3.44 +/− 0.13 SEM) (*p* = 0.994) and “negative impact” (riders: 4.29 +/− 0.07 SEM; dog owners: 4.31 +/− 0.08 SEM) (*p* = 0.869) were observed between riders and dog owners [63]. 

### 3.5. Association between Nature Relatedness and Wellbeing

Overall wellbeing (FAHW12) was significantly higher in recreational horseback riders compared to people without pets (*p* = 0.002), but no significantly higher scores compared to dog owners were found (*p* = 0.071). The evaluation of sub-scales shows significantly higher scores in riders for physical wellbeing compared to dog owners (*p* = 0.024) and people without pets (*p* = 0.006). The psychological aspect showed no significant difference between riders and dog owners (*p* = 0.587), but significantly higher levels compared to people without pets (*p* = 0.004). In social aspects of wellbeing (FAHW12), no significantly higher levels of wellbeing of riders compared to dog owners (*p* = 0.427) and people without pets (*p* = 0.317) were found. 

The correlation between overall wellbeing (FAHW 12) and nature relatedness was statistically non-significant in recreational horseback riders as well as in dog owners and people without pets. 

However, a significant correlation of physical wellbeing (FAHW12) with overall nature relatedness in horseback riders (*R* = 0.249, *p* = 0.042) and dog owners (*R* = 0.395, *p* = 0.003), but no significant correlation in people without pets (*R* = −0.070, *p* = 0.624), was found. Self-reported physical wellbeing correlated with sub-score nature experience in dog owners (*R* = 0.392, *p* = 0.003), but not in recreational horseback riders (*R* = 0.106, *p* = 0.391) and people without pets (*R* = 0.172, *p* = 0.222). Physical wellbeing of dog owners also showed a significant correlation with nature self (*R* = 0.282, *p* = 0.035) and nature perspective scores (*R* = 0.274, *p* = 0.041). 

Psychological and social wellbeing (FAHW 12) showed no significant correlation with nature relatedness. Additionally, no correlation of self-reported physical, psychological or social wellbeing and nature relatedness scores and sub-scores during and after the activity in recreational horseback riders was found.

### 3.6. Association between Mood Barometer and Nature Relatedness

The mood barometer (assessment of happiness) during/after activity shows differences between the groups. Overall, 79% of recreational horseback riders rated their feeling as very good (A or B), 70% of dog owners and 59% of people without pets did so; 21% of riders, 28% of dog owners and 41% of people without pets rated their feelings as average (Category C or D). Negative feeling during or after the activity was only present in one dog owner (Table 2).

Mood ratings differ between groups. A significantly better mood was found in recreational horseback riders compared to people without pets (*p* = 0.034), but this was similar compared to dog owners (*p* = 0.339). 

Mood ratings and NR-self correlated in recreational horseback riders (*R* = 0.250, *p* = 0.042) and dog owners showed a correlation between mood ratings and overall NR (*R* = 0.283, *p* = 0.038) and NR perspective (*R* = 0.303, *p* = 0.026).

The duration of positive feelings and subscore nature experience correlated significantly in riders (*R* = 0.289, *p* = 0.042) and people without pets (*R* = 0.352, *p* = 0.024); however, no correlation was found in dog owners (*R* = 0.139, *p* = 0.363).

### 3.7. Structural Equation Modeling

**Figure 2:**
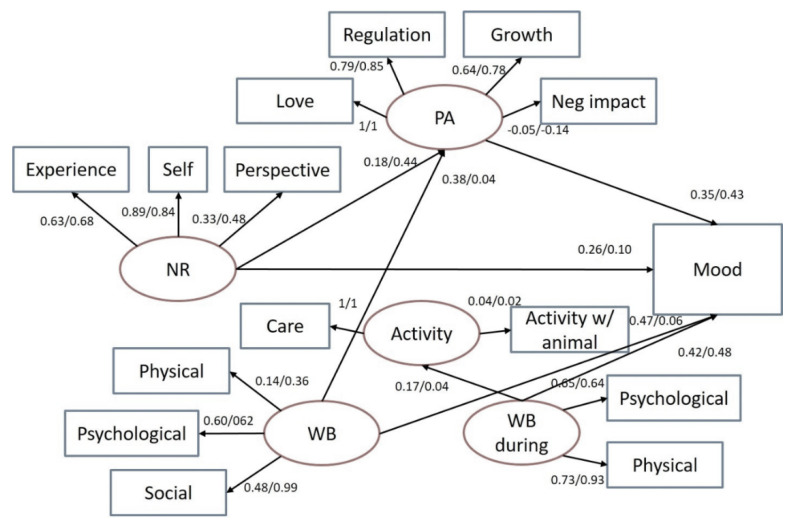
Structural equation model, standardized coefficients for riders/dog owners with latent variables in ellipses and observed variables in rectangles.

Figure 2 shows the structural equation model applied to nature relatedness (NR), pet attachment (PA) and wellbeing (WB) and mood ratings. Only riders and dog owners were included in this analysis because we had not assessed pet attachment in controls. 

Nature relatedness of riders revealed a direct path to self-reported mood (0.26) as distinctly higher compared to dog owners (0.10). In contrast, dog owners showed an indirect relationship between NR and mood via pet attachment (0.44) that was more pronounced in this group compared to riders (0.18). Furthermore, pet attachment was more strongly related to mood in dog owners (0.43) compared to riders (0.35). As can be seen, overall wellbeing was more strongly related to pet attachment in riders (0.38) compared to dog owners (0.04) and also to mood (riders: 0.47, dog owners: 0.06). Wellbeing during the activity showed a stronger relationship with physical activity in riders (0.17) versus dog owners (0.04). By contrast, wellbeing during the activity had nearly the same relationship with mood in riders (0.65) compared to dog owners (0.64) (Figure 2). 

## 4. Discussion

The present study combined attachment to nature, animal bonding, and physical activity with wellbeing and mood of middle- and older-aged (45+) horseback riders in comparison with dog owners and people without pets as controls. 

We showed for the first time that both riders and dog owners profit in wellbeing and mood from their pets, but the structure of these relationships differs.

### 4.1. Nature Relatedness and its Impact on Wellbeing

Riders had significantly higher nature relatedness compared to people without pets but were similar to dog owners, which also showed higher scores than people without pets (Table 1). Dog owners often live near green spaces to walk their dogs [86,87], and nearby nature is associated with psychological and social health benefits [31], but we have no evidence that they also have high nature relatedness.

Community gardening in the elderly [48] and children [49] can lead to physical and social benefits, but no differences in health and wellbeing can be found for the elderly compared to younger gardeners [50]. 

Similarly, in recreational horseback riders, significantly higher self-reported overall wellbeing (FAHW12) compared to people without pets similar to dog owners could be seen.

Riders reported a significantly higher score of physical wellbeing compared to dog owners and people without pets, which was in accordance with other studies. Riding is associated with increased trunk stabilization [9] and muscle strengthening [7,8], which might explain this difference.

In psychological wellbeing, similar levels of riders compared to dog owners, but significantly higher levels compared to people without pets, were shown. Natural environments are associated with positive feelings [39,40], and decreased depression [28], higher perceived mental [88] health [35], indicating that activity in nature alone may explain our findings. Furthermore, contact with nature and even images of natural environments can help to decrease stress and anxiety and also supports recovery from illness [78].

The social aspect of wellbeing (FAHW 12) showed no differences in the wellbeing of riders compared to dog owners and compared to people without pets. This was in contrast to studies, which report that dog walking induces social interactions [19,89,90] and regular meetings with other dog owners [91]. Interaction with animals increases motivation and leads to social advantages [92], effects we could not corroborate.

Concerning the association between relatedness to nature and wellbeing [31,78], our results indicate that this relationship is not necessarily a direct one but could—especially in dog owners—be mediated by pet attachment if experience in nature is motivated by activities with the pet (Figure 2). 

Physical wellbeing (FAHW12) correlated with overall nature relatedness in horseback riders and dog owners, whereas no correlation was found in people without pets. However, in contrast to studies reporting positive correlations between relatedness to nature and vitality and positive mood [28,47] we found a direct association in riders only, while in dog owners this relationship was mediated by pet attachment (Figure 2). Leisure time activities in green spaces are associated with a lower risk of mental health problems [29,93], and depression [28,45].

We found significantly better self-reported mood ratings in recreational horseback riders compared to people without pets, but similar in comparison to dog owners (Table 2). These mood ratings correlated with sub-scores of nature relatedness, in contrast to people without pets, both in riders and dog owners. Additionally, the duration of positive feelings of riders correlated with nature experience. This indicates that during riding, a joy in their life and also self-acceptance [78] might be improved by this activity.

Consistent with other studies, our results showed higher life satisfaction [33,34], higher happiness [26] and wellbeing [32] in people who reported a higher connectedness to nature. 

### 4.2. Physical Activity

The three groups were comparable concerning their daily physical activities, since we found no significant difference in sports and household activities between the three groups. However, we observed higher overall activity scores in riders and dog owners after the inclusion of pet-related activities, such as riding, dog playing and accompanying activities. Activities with pets, such as horseback riding and dog playing, provides the possibility to reach the recommended activity levels even at an older age (for adults 150 min of moderate intensity aerobic exercise per week or 500–1000 MET-minutes per week) [83,94,95]. In addition to the activity levels, higher psychological wellbeing scores compared to people without pets were found.

### 4.3. Pet Attachment 

High pet attachment in pet owners has been reported [15,55,56,96]. Our study showed lower scores in horseback riders in the “love” factor and “regulation of emotions” factor compared to dog owners. People without animals were not interviewed and therefore not evaluated [63]. The reason for these findings may be found in the living circumstances of dog owners. Proximity to their pets over several hours per day may cause stronger emotional closeness. Horseback riders see their horses only a few hours per day or week because horse stables in general are further away from the homes of their owners. Dog owners have longer daily contact and may even sleep with their dogs, an impossibility for riders [63].

Both in riders and dog owners self-reported mood ratings during/after the activity correlated with their pet attachment [63], which was in accordance with other studies [20,97]. They feel positive emotions [97] and happiness [20]. 

### 4.4. Structural Equation Model

The structural equation model demonstrates the direct pathway between nature relatedness to self-reported mood in riders, whereas in dog owners, this relationship is indirectly mediated by pet attachment. We suggested that riding in a nature environment and mood influenced each other, but that dog owners have a connection to nature because of their love for their animals. Moreover, overall wellbeing is more strongly related to pet attachment in riders compared to dog owners and also to mood (Figure 2). This indicates that during riding, wellbeing, positive emotions and happiness are improved by this activity. In contrast, wellbeing during the activity shows a similar relationship to mood, comparing riders and dog owners, but a stronger association between physical activity and mood was found in riders compared to dog owners (Figure 2). This indicates that riding could be seen as moderate physical activity, which affects mood.

### 4.5. Limitations of the Study

Our study has several limitations. Due to the study design as a cross-sectional study, no causal conclusions can be drawn. Nevertheless, the relationships extracted from the data should be considered as supporting recommendations within a program to increase physical activity in older-aged people in natural environments. Future studies should adopt a longitudinal design to examine changes in wellbeing and happiness, of pet attachment and nature relatedness over time. Another limitation of this study is that it has been conducted in individuals 45+ years of age from Austria. Thus, studies in other countries or different age groups are warranted. However, studies indicate no differences in health and wellbeing in the elderly [48], younger people [50] and children [49] due to leisure time working in nature. Moreover, activity levels are positively influenced by dog walking in children [14,15], in adults [16,17] and also in the elderly [18,19,20]. Additionally, high levels of pet attachment are shown in children [56], in adults [55], and in elderly people [20,58,59]. Another limitation should be mentioned that due to the recruitment strategy, a selection bias was possible. Further, our study was based on questionnaires. The participation rate was low, but the response rate was high, which could also have led to self-selection bias. Another limitation was that we did not inquire into household size, which could have been relevant concerning pet attachment because singles living with a pet could be very different from those who have a family. 

Nevertheless, to our knowledge, this is the first study which addressed the relationship between recreational horseback riding, pet attachment, nature relatedness, physical activity and wellbeing in older people so far. This could be important for future endeavors to improve the quality of life in an aging population.

## 5. Conclusions

We could find support for our first hypothesis that riders have similar levels of activity, overall NR scores and wellbeing compared to dog owners. Consistent with our second hypothesis that NR scores, pet attachment and activity levels influence wellbeing and happiness (mood), we observed a pattern of such relationships both in riders and dog owners that is similar but also shows some differences that should be further explored. 

This study adds additional evidence and extends existing knowledge about nature relatedness, pet attachment and activities with dogs and horses. Pet ownership, regardless of whether horses or dogs, promotes wellbeing and happiness by activities with and closeness to these animals [63]. Additionally, horseback riding and dog walking might help to reach the recommended activity levels. The results suggest that horseback riding provides similar benefits as are already known for dog walking. 

## Figures and Tables

**Table 1:** Adjusted nature relatedness-score (mean +/− SEM) and associated *p*-values of nature relatedness overall scale and sub-scales.

Scale	Riders (1)	Dog Owners (2)	Without Pets (3)	*p*-Value (1)–(2)	*p*-Value (1)–(3)
Overall	4.13 +/− 0.06	4.04 +/− 0.06	3.69 +/− 0.07	0.325	≤0.001
Nature experience	3.84 +/− 0.09	3.82 +/− 0.09	3.24 +/− 0.1	0.878	≤0.001
Nature self	4.33 +/− 0.07	4.23 +/− 0.07	4.05 +/− 0.08	0.338	0.018
Nature perspective	4.16 +/− 0.07	4.02 +/− 0.08	3.65 +/− 0.08	0.234	≤0.001

**Table 2:** Mood estimation.

Mood Rating		Riders	Dog Owners	Without Pets
Very good (AB)	Frequency	53	38	30
	Frequency in %	79.1%	70.4%	58.8%
Good (CD)	Frequency	14	15	21
	Frequency in %	20.9%	27.8%	41.2%
Bad (EF)	Frequency	0	1	0
	Frequency in %	0.0%	1.9%	0.0%
Very bad (G)	Frequency	0	0	0
	Frequency in %	0.0%	0.0%	0.0%
%, Percent				

## References

[1] McPhee J.S., French D.P., Jackson D., Nazroo J., Pendleton N., Degens H. (2016). Physical activity in older age: Perspectives for healthy ageing and frailty. Biogerontology.

[2] Warr P., Butcher V., Robertson I. (2004). Activity and psychological well-being in older people. Aging Ment. Health.

[3] Pedersen M.T., Vorup J., Nistrup A., Wikman J.M., Alstrøm J.M., Melcher P.S., Pfister G.U., Bangsbo J. (2017). Effect of team sports and resistance training on physical function, quality of life, and motivation in older adults. Scand. J. Med. Sci. Sports.

[4] Rodríguez-Gómez I., Mañas A., Losa-Reyna J., Rodríguez-Mañas L., Chastin S.F.M., Alegre L.M., García-García F.J., Ara I. (2018). Associations between sedentary time, physical activity and bone health among older people using compositional data analysis. PLoS ONE.

[5] Lohne-Seiler H., Kolle E., Anderssen S.A., Hansen B.H. (2016). Musculoskeletal fitness and balance in older individuals (65–85 years) and its association with steps per day: A cross sectional study. BMC Geriatr..

[6] Lechner M. (2009). Long-run labour market and health effects of individual sports activities. J. Health Econ..

[7] Koca T.T. (2016). What is hippotherapy? The indications and effectiveness of hippotherapy. North. Clin. Istanb..

[8] Cho S.-H. (2017). Effects of horseback riding exercise on the relative alpha power spectrum in the elderly. Arch. Gerontol. Geriatr..

[9] Kang K.-Y. (2015). Effects of mechanical horseback riding on the balance ability of the elderly. J. Phys. Ther. Sci..

[10] Vail J.D. (2009). Polo for all ages: Exercise should be functional and fun!. J. Psychosoc. Nurs. Ment. Health Serv..

[11] Rasciute S. (2010). Health or happiness? What is the impact of physical activity on the individual?. KYKLOS.

[12] World Health Organization (2018). Physical Activity: Fact Sheet (Accessed on 2 April 2018).

[13] Christian H.E., Westgarth C., Bauman A., Richards E.A., Rhodes R.E., Evenson K.R., Mayer J.A., Thorpe R.J. (2013). Dog ownership and physical activity: A review of the evidence. J. Phys. Act. Health.

[14] Westgarth C., Boddy L.M., Stratton G., German A.J., Gaskell R.M., Coyne K.P., Bundred P., McCune S., Dawson S. (2016). The association between dog ownership or dog walking and fitness or weight status in childhood. Pediatr. Obes..

[15] Westgarth C., Boddy L.M., Stratton G., German A.J., Gaskell R.M., Coyne K.P., Bundred P., McCune S., Dawson S. (2013). A cross-sectional study of frequency and factors associated with dog walking in 9-10 year old children in Liverpool, UK. BMC Public Health.

[16] Mein G., Grant R. (2018). A cross-sectional exploratory analysis between pet ownership, sleep, exercise, health and neighbourhood perceptions: The Whitehall II cohort study. BMC Geriatr..

[17] Westgarth C., Liu J., Heron J., Ness A.R., Bundred P., Gaskell R.M., German A.J., McCune S., Dawson S., Thorne C. (2012). Dog Ownership during pregnancy, maternal activity, and obesity: A cross-sectional study. PLoS ONE.

[18] Ruzić A., Miletić B., Ruzić T., Persić V., Laskarin G. (2011). Regular dog-walking improves physical capacity in elderly patients after myocardial infarction. Coll. Antropol..

[19] Toohey A.M., McCormack G.R., Doyle-Baker P.K., Adams C.L., Rock M.J. (2013). Dog-walking and sense of community in neighborhoods: Implications for promoting regular physical activity in adults 50 years and older. Health Place.

[20] Raina P., Waltner-Toews D., Bonnett B., Woodward C., Abernathy T. (1999). Influence of companion animals on the physical and psychological health of older people: An analysis of a one-year longitudinal study. J. Am. Geriatr. Soc..

[21] Nisbet E.K., Zelenski J.M., Murphy S.A. (2009). The nature relatedness scale: Linking individuals’ connection with nature to environmental concern and behavior. Environ. Behav..

[22] Zelenski J.M., Nisbet E.K. (2014). Happiness and feeling connected: The distinct role of nature relatedness. Environ. Behav..

[23] Wilson E.O. (1984). Biophilia.

[24] Melson L.G. (2005). Why the Wild Things Are. Animals in the Lives of Children.

[25] Kellert S.R. (1997). Kinship to Mastery. Biophilia in Human Evolution and Development.

[26] Capaldi C.A., Dopko R.L., Zelenski J.M. (2014). The relationship between nature connectedness and happiness: A meta-analysis. Front. Psychol..

[27] Shanahan D.F., Lin B.B., Bush R., Gaston K.J., Dean J.H., Barber E., Fuller R.A. (2015). Toward improved public health outcomes from urban nature. Am. J. Public Health.

[28] Shanahan D.F., Bush R., Gaston K.J., Lin B.B., Dean J., Barber E., Fuller R.A. (2016). Health benefits from nature experiences depend on dose. Sci. Rep..

[29] Lawton E., Brymer E., Clough P., Denovan A. (2017). The relationship between the physical activity environment, nature relatedness, anxiety, and the psychological well-being benefits of regular exercisers. Front. Psychol..

[30] Kingsley J.Y., Townsend M., Henderson-Wilson C. (2009). Cultivating health and wellbeing: Members’ perceptions of the health benefits of a Port Melbourne community garden. Leis. Stud..

[31] Cox D.T.C., Shanahan D.F., Hudson H.L., Fuller R.A., Anderson K., Hancock S., Gaston K.J. (2017). Doses of nearby nature simultaneously associated with multiple health benefits. Int. J. Environ. Health Res..

[32] Cervinka R., Roderer K., Hefler E. (2012). Are nature lovers happy? On various indicators of well-being and connectedness with nature. J. Health Psychol..

[33] Swami V., Barron D., Weis L., Furnham A. (2016). Bodies in nature: Associations between exposure to nature, connectedness to nature, and body image in U.S. adults. Body Image.

[34] Zelenski J.M., Dopko R.L., Capaldi C.A. (2015). Cooperation is in our nature: Nature exposure may promote cooperative and environmentally sustainable behavior. J. Environ. Psychol..

[35] Puhakka S., Pyky R., Lankila T., Kangas M., Rusanen J., Ikäheimo T.M., Koivumaa-Honkanen H., Korpelainen R. (2018). Physical activity, residential environment, and nature relatedness in young men-A population-based MOPO study. Int. J. Environ. Health Res..

[36] Isaacs A., Critchley J., See Tai S., Buckingham K., Westley D., Harridge S., Smith C., Gottlieb J. (2007). Exercise evaluation randomised trial (EXERT): A randomised trial comparing GP referral for leisure centre-based exercise, community-based walking and advice only. Health Technol. Assess.

[37] Pasanen T.P., Tyrväinen L., Korpela K.M. (2014). The relationship between perceived health and physical activity indoors, outdoors in built environments, and outdoors in nature. Appl. Psychol. Health Well-Being.

[38] Li Q., Morimoto K., Kobayashi M., Inagaki H., Katsumata M., Hirata Y., Hirata K., Suzuki H., Li Y.J., Wakayama Y. (2008). Visiting a forest, but not a city, increases human natural killer activity and expression of anti-cancer proteins. Int. J. Immunopathol. Pharmacol..

[39] Tsunetsugu Y., Park B.-J., Ishii H., Hirano H., Kagawa T., Miyazaki Y. (2007). Physiological effects of Shinrin-yoku (taking in the atmosphere of the forest) in an old-growth broadleaf forest in Yamagata Prefecture, Japan. J. Physiol. Anthropol..

[40] Park B.J., Tsunetsugu Y., Kasetani T., Kagawa T., Miyazaki Y. (2010). The physiological effects of Shinrin-yoku (taking in the forest atmosphere or forest bathing): Evidence from field experiments in 24 forests across Japan. Environ. Health Prev. Med..

[41] Li Q., Morimoto K., Kobayashi M., Inagaki H., Katsumata M., Hirata Y., Hirata K., Shimizu T., Li Y.J., Wakayama Y. (2008). A forest bathing trip increases human natural killer activity and expression of anti-cancer proteins in female subjects. J. Biol. Regul. Homeost. Agents.

[42] Mayer F.S., Frantz C.M. (2004). The connectedness to nature scale: A measure of individuals’ feeling in community with nature. J. Environ. Psychol..

[43] Bowler D.E., Buyung-Ali L.M., Knight T.M., Pullin A.S. (2010). A systematic review of evidence for the added benefits to health of exposure to natural environments. BMC Public Health.

[44] Mayer F.S., Frantz C.M., Bruehlman-Senecal E., Dolliver K. (2009). Why is nature beneficial?: The role of connectedness to nature. Environ. Behav..

[45] Thompson Coon J., Boddy K., Stein K., Whear R., Barton J., Depledge M.H. (2011). Does participating in physical activity in outdoor natural environments have a greater effect on physical and mental wellbeing than physical activity indoors? A systematic review. Environ. Sci. Technol..

[46] Maier J., Jette S. (2016). Promoting Nature-based activity for people with mental illness through the US “Exercise Is Medicine” initiative. Am. J. Public Health.

[47] Berman M.G., Kross E., Krpan K.M., Askren M.K., Burson A., Deldin P.J., Kaplan S., Sherdell L., Gotlib I.H., Jonides J. (2012). Interacting with nature improves cognition and affect for individuals with depression. J. Affect. Disord..

[48] Scott T.L., Masser B.M., Pachana N.A. (2020). Positive aging benefits of home and community gardening activities: Older adults report enhanced self-esteem, productive endeavours, social engagement and exercise. Sage Open Med..

[49] Eng S., Khun T., Jower S., Murro M.J. (2019). Healthy Lifestyle Through Home Gardening: The Art of Sharing. Am. J. Lifestyle Med..

[50] Van den Berg A.E., van Winsum-Westra M., de Vries S., van Dillen S.M.E. (2010). Allotment gardening and health: A comparative survey among allotment gardeners and their neighbors without an allotment. Environ. Health.

[51] Bowlby J. (1975). Bindung. Eine Analyse der Mutter-Kind-Beziehung.

[52] Beetz A., Julius H., Turner D., Kotrschal K. (2012). Effects of social support by a dog on stress modulation in male children with insecure attachment. Front. Psychol..

[53] Fine A.H. (2010). Handbook on Animal-Assisted Therapy. Theoretical Foundations and Guidelines for Practice.

[54] Asselin G., Penning J.H., Ramanujam S., Neri R., Ward C. (2015). Therapeutic horse back riding of a spinal cord injured veteran: A case study. Rehabil. Nurs..

[55] Freiwald A., Litster A., Weng H.-Y. (2014). Survey to investigate pet ownership and attitudes to pet care in metropolitan Chicago dog and/or cat owners. Prev. Vet. Med..

[56] Hawkins R.D., Williams J.M., Scottish Society for the Prevention of Cruelty to Animals (Scottish SPCA) (2017). Childhood attachment to pets: Associations between pet attachment, attitudes to animals, compassion, and humane behaviour. Int. J. Environ. Health Res..

[57] Hegedusch E., Hegedusch L. (2007). Tiergestützte Therapie bei Demenz. Die Gesundheitsförderliche Wirkung von Tieren auf Demenziell Erkrankte Menschen.

[58] Kruger K.S., Stern S.L., Anstead G., Finley E.P. (2014). Perceptions of companion dog benefits on well-being of US military veterans with HIV/AIDS. South. Med. J..

[59] Mueller M.K., Gee N.R., Bures R.M. (2018). Human-animal interaction as a social determinant of health: Descriptive findings from the health and retirement study. BMC Public Health.

[60] Smolkovic I. (2012). Attachment to pets and interpersonal relationships. J. Eur. Psychol. Stud..

[61] Winefield H.R., Black A., Chur-Hansen A. (2008). Health effects of ownership of and attachment to companion animals in an older population. Int. J. Behav. Med..

[62] Kotrschal K. (2016). Hund & Mensch. Das Geheimnis unserer Seelenverwandtschaft.

[63] Schwarzmueller-Erber G., Maier M., Kundi M. (2020). Pet attachment and wellbeing of older-aged recreational horseback riders. Int. J. Environ. Health Res..

[64] Gadomski A.M., Scribani M.B., Krupa N., Jenkins P. (2017). Pet dogs and child physical activity: The role of child-dog attachment. Pediatr. Obes..

[65] Shibata A., Oka K., Inoue S., Christian H., Kitabatake Y., Shimomitsu T. (2012). Physical activity of Japanese older adults who own and walk dogs. Am. J. Prev. Med..

[66] Wood L., Martin K., Christian H., Nathan A., Lauritsen C., Houghton S., Kawachi I., McCune S. (2015). The pet factor--companion animals as a conduit for getting to know people, friendship formation and social support. PLoS ONE.

[67] Flegr J., Preiss M. (2019). Friends with malefit. The effects of keeping dogs and cats, sustaining animal-related injuries and Toxoplasma infection on health and quality of life. PLoS ONE.

[68] Brooks H.L., Rushton K., Lovell K., Bee P., Walker L., Grant L., Rogers A. (2018). The power of support from companion animals for people living with mental health problems: A systematic review and narrative synthesis of the evidence. BMC Psychiatry.

[69] Friedman E., Krause-Parello C.A. (2018). Companion animals and human health: Benefits, challenges, and the road ahead for human-animal interaction. Rev. Off. Int. Epizoot..

[70] Schröter C., Schulte-Sutum A., Zeckey C., Winkelmann M., Krettek C., Mommsen P. (2017). Unfälle im Reitsport: Analyse von Verletzungsmechanismen und mustern. Unfallchirurg.

[71] Van Balen P.-J., Barten D.G., Janssen L., Fiddelers A.A.A., Brink P.R., Janzing H.M.J. (2019). Beware of the force of the horse: Mechanisms and severity of equestrian-related injuries. Eur. J. Emerg. Med..

[72] Stevens J.A., Teh S.L., Haileyesus T. (2010). Dogs and cats as environmental fall hazards. J. Saf. Res..

[73] Willmott H., Greenheld N., Goddard R. (2012). Beware of the dog? An observational study of dog-related musculoskeletal injury in the UK. Accid. Anal. Prev..

[74] Cherniack E.P., Cherniack A.R. (2015). Assessing the benefits and risks of owning a pet. CMAJ.

[75] Illek S., Winkler D. Österreichischer Pferdesportverband-20 Jahre im Statistischen Überblick: Tabellarische und Grafische Auswertungen der EDV-Daten des OEPS von 1999 bis 2018. http://www.oeps.at/main.asp?VID=1&kat1=87&kat2=574&Text=&DMKID=215.

[76] Huschka A. Der österreichische Kynologenverband (ÖKV). https://oekv.at/de/oekv/oesterr-kynologenverband.

[77] World Health Organization (2001). Declaration of Helsinki. Bulletin of the World Health Organization.

[78] Nisbet E.K., Zelenski J.M., Murphy S.A. (2011). Happiness is in our nature: Exploring nature relatedness as a contributor to subjective well-being. J. Happiness Stud..

[79] Cromer L.D.M., Barlow M.R. (2013). Factors and convergent validity of the pet attachment and life impact scale (PALS). Hum. Anim. Interact. Bull..

[80] Wydra G. (2014). Der Fragebogen zum Allgemeinen Habituellen Wohlbefinden.

[81] Kunin T. (1955). The construction of a new type of attitude measure. Pers. Psychol..

[82] Andrews F.M., Withey S.B. (1978). Social Indicators of Well Being. Americans Perceptions’ of Life Quality.

[83] Ainsworth B.E., Haskell W.L., Herrmann S.D., Meckes N., Bassett J.R., Tudor-Locke C., Greer J.L., Vezina J., Whitt-Glover M.C., Leon A.S. (2018). The Compendium of Physical Activities Tracking Guide (Accessed on 21 March 2018).

[84] Lilliefors H.W. (1967). On the Kolmogorov-Smirnov test for normality with mean and variance Unknown. J. Am. Stat. Assoc..

[85] Levene H. (1960). Robust Tests for Equality of Variances.

[86] Coleman K.J., Rosenberg D.E., Conway T.L., Sallis J.F., Saelens B.E., Frank L.D., Cain K. (2008). Physical activity, weight status, and neighborhood characteristics of dog walkers. Prev. Med..

[87] Westgarth C., Christley R.M., Christian H.E. (2014). How might we increase physical activity through dog walking?: A comprehensive review of dog walking correlates. Int. J. Behav. Nutr. Phys. Act..

[88] Sugiyama T., Leslie E., Giles-Corti B., Owen N. (2008). Associations of neighbourhood greenness with physical and mental health: Do walking, social coherence and local social interaction explain the relationships?. J. Epidemiol. Commun. Health.

[89] Cutt H., Giles-Corti B., Knuiman M. (2008). Encouraging physical activity through dog walking: Why don’t some owners walk with their dog?. Prev. Med..

[90] Feng Z., Dibben C., Witham M.D., Donnan P.T., Vadiveloo T., Sniehotta F., Crombie I.K., McMurdo M.E.T. (2014). Dog ownership and physical activity in later life: A cross-sectional observational study. Prev. Med..

[91] Heuberger R., Wakshlag J. (2011). Characteristics of ageing pets and their owners: Dogs v. cats. Br. J. Nutr..

[92] Taniguchi Y., Seino S., Nishi M., Tomine Y., Tanaka I., Yokoyama Y., Ikeuchi T., Kitamura A., Shinkai S. (2019). Association of Dog and Cat Ownership with Incident Frailty among Community-Dwelling Elderly Japanese. Sci. Rep..

[93] Mitchell R. (2013). Is physical activity in natural environments better for mental health than physical activity in other environments?. Soc. Sci. Med..

[94] Titze S., Ring-Dimitriou S., Schober P.H., Halbwachs C., Samitz G., Miko H.C., Lercher P., Stein K.V., Gäbler C., Bauer R. Österreichische Empfehlungen für Gesundheitswirksame Bewegung. http://fgoe.org/sites/fgoe.org/files/2017-10/2012-10-17.pdf.

[95] Parger A. (2009). Bewegung. Jeder Schritt Zählt.

[96] Rodriguez K.E., Bibbo J., O’Haire M.E. (2019). The effects of service dogs on psychosocial health and wellbeing for individuals with physical disabilities or chronic conditions. Disabil. Rehabil..

[97] Kerns K.A., Stuart-Parrigon K.L., Coifman K.G., van Dulmen M.H.M., Koehn A. (2018). Pet Dogs: Does their presence influence preadolescents’ emotional responses to a social stressor?. Soc. Dev..

